# Culture conversion and macrolide resistance in *Mycobacterium abscessus* complex pulmonary disease

**DOI:** 10.1128/spectrum.01274-25

**Published:** 2025-09-02

**Authors:** Suting Chen, Shengjuan Bao, Jifang Zheng, Guanglu Jiang, Hongfei Duan, Hairong Huang

**Affiliations:** 1 Beijing Key Laboratory for Drug-Resistant Tuberculosis Research, National Clinical Laboratory on Tuberculosis, Beijing Chest Hospital, affiliated Capital Medical University, Beijing Tuberculosis and Thoracic Tumor Institute 12517, Beijing, China; 2 Tuberculosis Department, Beijing Chest Hospital, affiliated Capital Medical University, Beijing Tuberculosis and Thoracic Tumor Institute 12517, Beijing, China; University of Pittsburgh School of Medicine, Pittsburgh, Pennsylvania, USA

**Keywords:** macrolides, inducible resistance, *Mycobacterium abscessus *complex, culture conversion

## Abstract

The *Mycobacterium abscessus* complex (MABC) is highly resistant to antibiotics, making the pulmonary disease it causes, known as MABC-PD, difficult to treat. Macrolide antibiotics are the main treatment for MABC-PD, but there is ongoing debate about whether to favor azithromycin (AZI) or clarithromycin (CLA). We compared how well AZI and CLA work against MABC and monitored the risk of developing resistance. MABC-PD patients were enrolled, and their primary isolates were collected. The minimum inhibitory concentrations (MICs) of CLA and AZI against these isolates over various incubation times were determined. The incidences of the acquired and inducible resistance of the isolates to both antibiotics were compared, and their association with culture conversion was monitored. The transcription levels of the *erm*(41) gene were quantified. A total of 61 MABC-PD patients who underwent a macrolide-containing treatment regimen were enrolled. The MICs of AZI against the MABC clinical isolates were found to be approximately four to eight times higher than those of CLA. Inducible macrolide resistance was observed in the subspecies *Mycobacterium abscessus*, which possessed the functional *erm*(41) gene. Inducible resistance to AZI happened more rapidly than to CLA, and the transcription of the *erm*(41) elevated significantly after prolonged exposure to macrolides. Patients infected with isolates with functional *erm(41*) gene or *rrl* mutation experienced lower rates of culture conversion compared to those without such mutations. Acquired and inducible resistance to macrolides significantly contributes to the treatment failures of MABC-PD. The *in vitro* sensitivity and the inducible resistance occurrence all favor CLA over AZI for MABC-PD treatment.

**IMPORTANCE:**

The *Mycobacterium abscessus* complex (MABC) is highly resistant to antibiotics, making *Mycobacterial abscessus* pulmonary disease (MAPD) difficult to treat. Macrolide antibiotics are the main treatment, but there is debate over whether azithromycin (AZI) or clarithromycin (CLA) is more effective. In our study of 61 MAPD patients, we compared the effectiveness of AZI and CLA while tracking resistance. We found that the minimum inhibitory concentrations (MICs) of AZI were four to eight times higher than those of CLA. Isolates with the functional *erm*(41) gene exhibited macrolide resistance, with AZI developing resistance faster than CLA. Patients with these resistant strains had lower rates of successful treatment. Overall, the *in vitro* sensitivity and the inducible resistance occurrence all favor CLA over AZI for MABC-PD treatment.

## INTRODUCTION

Non-tuberculous mycobacterial (NTM) infections have increased in recent years, garnering considerable attention. The *Mycobacterium abscessus* complex (MABC), the most frequently isolated rapid-growing NTM in many countries, often causes lung and skin soft tissue infections (1, 2). MABC is notorious for its resistance to many conventional antibiotics, making infection management especially challenging, particularly in patients with underlying conditions, such as cystic fibrosis (3, 4). Effective strategies to combat MABC are desperately needed.

The treatment for pulmonary disease caused by MABC (MABC-PD) primarily follows the 2020 multi-society guidelines for NTM infections (5). These guidelines recommend a multi-drug combination therapy, with macrolides serving as the core medication. Despite the fact that at least three drugs are included in the regimen, it is still very challenging to achieve sustained sputum culture conversion (4, 6–8). Only about 30% of these MABC-PD patients can be cured. The MABC comprises three distinct subspecies: *Mycobacterium abscessus* subspecies *abscessus* (*M. abscessus*), *Mycobacterium abscessus* subspecies *massiliense* (*M. massiliense*), and *Mycobacterium abscessus* subspecies *bolletii* (*M. bolletii*) (9, 10). Specifically, *M. massiliense* has been associated with better treatment outcomes compared to *M. abscessus* (1, 6, 11–13).

Macrolide antibiotics, such as clarithromycin (CLA) and azithromycin (AZI), have been shown to exert their effects through direct bactericidal action or indirect immunomodulatory mechanisms (14, 15). However, the development of resistance against these antibiotics worsens the prognosis of the disease. Resistance to macrolides in MABC can be categorized into two main types: acquired and inducible resistance. Acquired resistance is often mediated by mutations in the *rrl* gene, which encodes 23S rRNA, at positions 2058/2059. This is a common mechanism for high-level resistance to macrolides, as these mutations can significantly reduce the binding affinity of macrolides to the bacterial ribosome, thereby diminishing their efficacy (15–17). Inducible resistance is typically associated with the expression level of the *erm*(41) gene (18). This gene can be upregulated in the presence of macrolides, leading to methylation of ribosomal RNA and subsequent resistance (7, 19). The different mechanisms that contribute to macrolide resistance make it difficult for clinicians to determine when to use macrolides to treat MABC-PD. In clinical practice, AZI was preferred over CLA because of better tolerance, fewer drug interactions, and equal efficacy with once-daily administration. Some studies showed that the level of *erm*(41) expression was higher after exposure to CLA than after exposure to AZI, resulting in a high level of inducible CLA resistance in *M. abscessus* isolates (8). Later, several other studies have suggested that AZI-induced resistance can occur earlier than with CLA (18) and that AZI also induces high levels of *erm*(41) expression (20). As a result, it is still controversial which commonly used macrolide antibiotics, AZI or CLA, is more beneficial for managing MABC diseases. The dynamic nature of resistance necessitates a careful consideration of antibiotic regimens to minimize the risk of resistance development and ensure effective treatment outcomes (21, 22).

This study aims to compare the antibacterial activities of AZI and CLA against MABC isolates *in vitro*, as well as to analyze the incidence of acquired and inducible resistance to AZI and CLA, providing valuable insights for the clinical treatment of MABC diseases.

## RESULTS

### MIC distribution of the MABC clinical isolates

A drug susceptibility test was conducted on primary isolates from 61 patients diagnosed with MABC-PD. Among these isolates, 36 were identified as *M. abscessus*, and the remaining were *M. massiliense*. No *M. bolletii* isolates were identified in our study. After incubating the isolates with CLA and AZI for 3 days, the MIC for all isolates was determined. For CLA, the MIC ranged from 0.0156 to >16 µg/mL, while for AZI, it ranged from 0.0625 to >64 µg/mL (see Table 1). The MIC levels for AZI were approximately four to eight times higher than those for CLA. In addition, the MIC_50_ and MIC_90_ values for AZI were significantly higher than those for CLA against MABC isolates. The MIC levels for the *M. abscessus* isolates were significantly higher than those of the *M. massiliense* isolates for both antibiotics. According to the resistance criteria established by the Clinical and Laboratory Standards Institute (CLSI) and existing literature (23, 24), 12 of the 36 *M*. *abscessus* isolates exhibited AZI resistance; 19 showed intermediate resistance; and five were susceptible to AZI. In contrast, none of the *M. abscessus* isolates were resistant to CLA; only one isolate demonstrated intermediate resistance. Additionally, only two *M*. *massiliense* isolates displayed resistance to both CLA and AZI at the third-day incubation, which was associated with mutations at positions 2058/2059 of the *rrl* gene. The remaining tested isolates of *M. massiliense* were found to be susceptible to both CLA and AZI.

**TABLE 1:** MIC value and resistance profile for CLA and AZI against the MABC isolates on the third day

Antibiotics	MIC range (μg/mL)	MIC_50_ (μg/mL)	MIC_90_ (μg/mL)	*M.abscessus* (*n* = 36)			*M.massiliense* (*n* = 25)		
				R	I	S	R	I	S
CLA^*a*^	0.0156–>16	0.03125	0.0625	0	1	35	2	0	23
AZI^*b*^	0.0625–>64	8	16	12	19	5	2	0	23

^*a*^ CLA = clarithromycin; b: AZI = azithromycin; S, susceptible; I, intermediate susceptible; and R, resistant.

^*b*^ MIC_50_: minimum concentration that can inhibit the growth of 50% of the tested bacteria in an *in vitro* drug sensitivity assay; MIC_90_: minimum concentration that can inhibit the growth of 90% of the tested bacteria in an *in vitro* drug sensitivity assay.

### Inducible macrolide resistance in MABC clinical isolates

**Fig 1:**
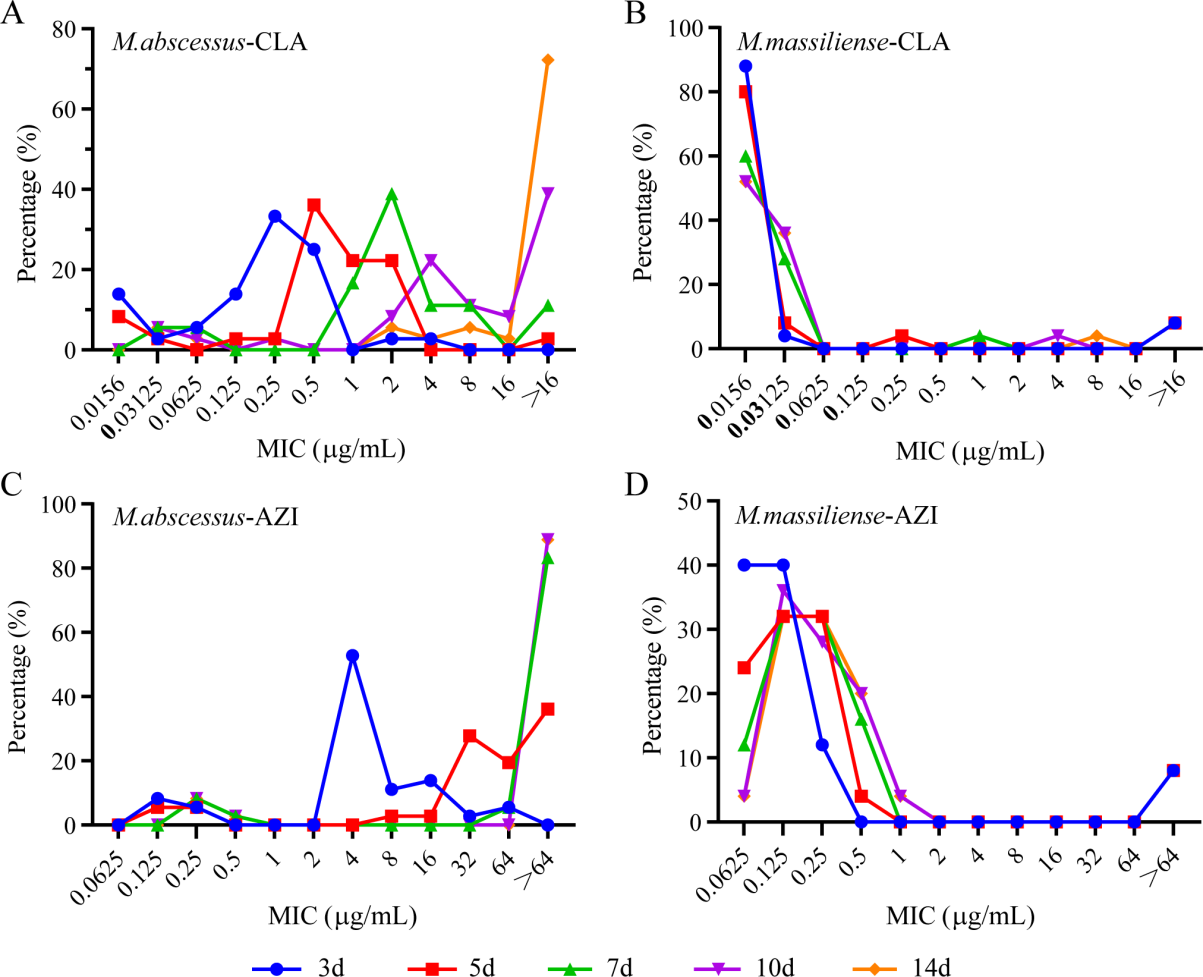
Inducible macrolide resistance in MABC clinical isolates at different incubation time points. (A) Induced CLA resistance in *M. abscessus*; (B) induced CLA resistance in *M. massiliense*; (C) induced AZI resistance in *M. abscessus*; and (D) induced AZI resistance in *M. massiliense*. The MIC values of CLA and AZI for the clinical isolates of various subspecies were measured at different time points of drug exposure (3, 5, 7, 10, and 14 days). The vertical axis indicates the proportion of strains with specific MIC values to the total number of tested strains.

As the incubation time in drug-containing media was increased, the MIC for nearly all *M. abscessus* isolates rose significantly (see Fig. 1; Fig. S1). After exposure to CLA and AZI, *M. abscessus* isolates exhibited different patterns of resistance. For CLA, resistance emerged gradually, with 3, 6, 16, and 29 isolates showing resistance on days 5, 7, 10, and 14, respectively. In contrast, AZI exhibited rapid early resistance, with 15 isolates developing resistance by day 5, peaking at 34 isolates by day 10, and no further increase observed by day 14 (Fig. 1A and C). Furthermore, AZI was found to induce resistance more frequently, with a rate of 55.7% (34/61), compared to 47.5% (29/61) for CLA at day 14 (*P* < 0.001). Meanwhile, *M. abscessus* isolates that develop inducible resistance to AZI earlier are also more likely to develop inducible resistance to CLA. In contrast, the MIC for *M. massiliense* isolates did not show any significant change with prolonged exposure to macrolides (Fig. 1B and D).

### Analysis of the *erm*(41) gene expression

**Fig 2:**
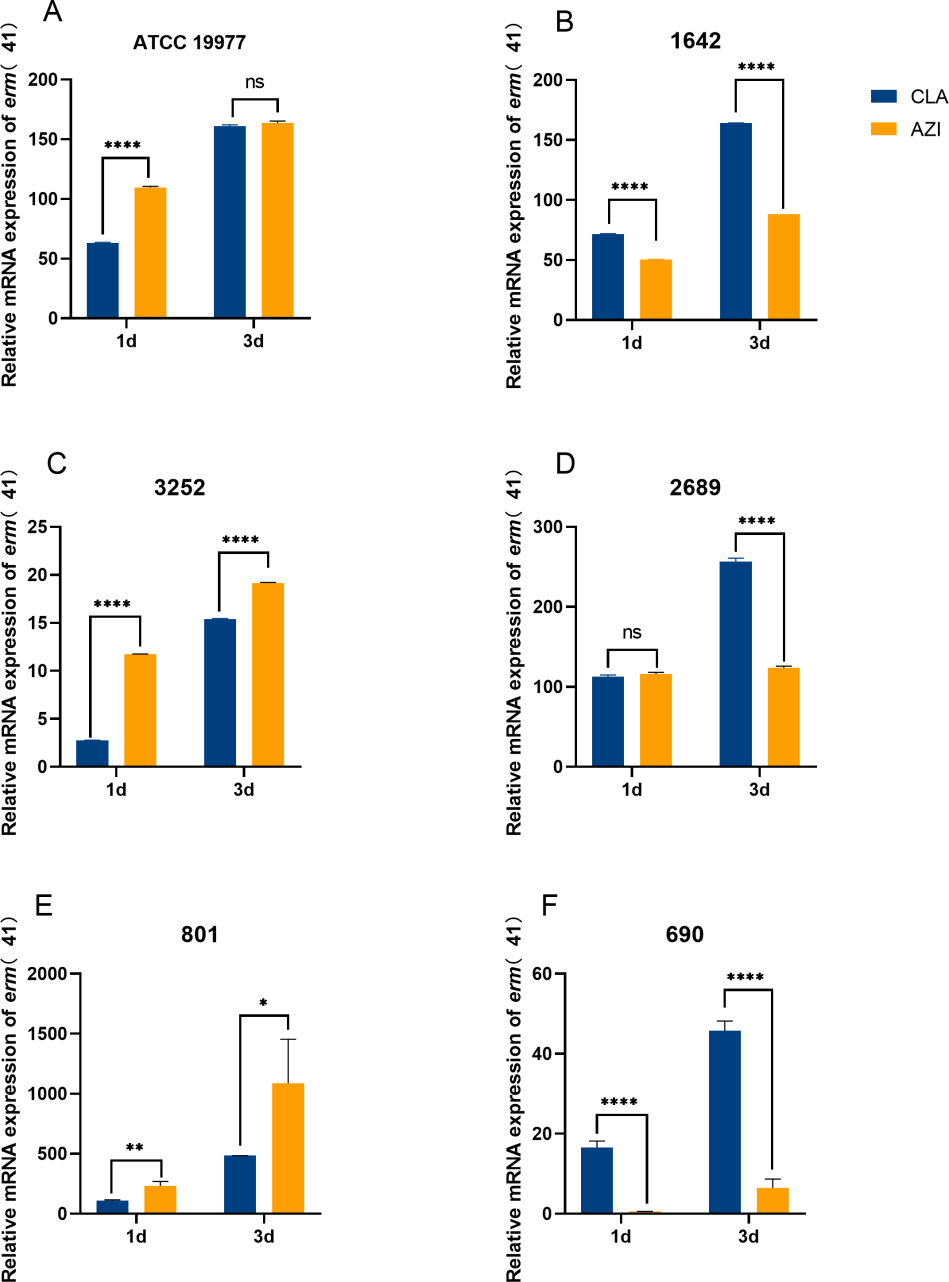
Relative mRNA expression of *erm*(41) induced by CLA and AZI in *M. abscessus* isolates with functional *erm*(41) gene on days 1 and 3. (A–F) Each part represents the transcriptional level of the *erm*(41) gene of a single strain. The relative mRNA level of *erm*(41) was calculated as the ratio of the drug exposure levels in the 1-and 3-day groups to that in the no-drug-exposure (0-day) group. The number at the top of each panel refers to the strain number. Data were presented as mean ± SD. Two-tailed *t*-tests were performed for the statistical analysis (*P < 0.05; **P < 0.01; ***P < 0.001; ****P < 0.0001; *****P < 0.00001).

The transcription levels of the *erm*(41) gene were analyzed after exposure to macrolides in six strains of *M. abscessus*, which included one reference strain and five clinical isolates, all possessing a functional *erm*(41) gene. All six strains developed inducible resistance to AZI, while two isolates did not exhibit inducible resistance to CLA (Table S1). After incubating the isolates with 1/2 MIC of each macrolide for either 24 or 72 h, the transcription levels of the *erm*(41) gene were assessed. We found that the transcription of the *erm*(41) gene was significantly elevated in response to both AZI and CLA exposures. Furthermore, the expression level of the *erm*(41) gene in half of the tested isolates treated with AZI was higher than that of the isolates treated with CLA, while the other half showed the opposite trend (Fig. 2). No significant correlation was found between the *erm*(41) gene expression levels under different drug exposures and the development of induced drug resistance for the corresponding drug.

### Sputum culture conversion

A total of 61 clinically diagnosed patients with MABC were enrolled, and their sputum culture conversion was analyzed retrospectively (see Table 2). Sputum conversion was achieved in 23 patients, accounting for 37.7% of the total (23/61). Statistical analysis showed no significant relationship between sputum culture conversion and patient gender (*P* = 0.610) or age (*P* = 0.682). Among the 36 patients infected with *M. abscessus*, the sputum conversion rate was 25% (9/36), which was significantly lower than that of *M. massiliense*, of which the sputum conversion rate was 56% (14/25) (*P* = 0.014).

**TABLE 2:** Univariate analysis of factors associated with sputum culture conversion in MABC-PD patients^*a*^

	Culture conversion	Non-conversion	*P* value
	*n* = 23	*n* = 38	
Baseline conditions			
Sex, female, no. (%)	16 (69.6)	24 (63.2)	0.610
Age, years, median (IQR)	52 (41.0–63.0)	49.5 (35.0–62.0)	0.682
Subspecies, no. (%)			
*M. abscessus*	9 (39.1)	27 (71.1)	0.014
*M. massiliense*	14 (60.9)	11 (28.9)	
Colony, no. (%)			
Rough	14 (60.9)	25 (65.8)	0.698
Smooth	9 (39.1)	13 (34.2)	
Induced drug resistance (Y/N), no. (%)			
CLA Y	6 (26.1)	23 (60.5)	0.009
CLA N	17 (73.9)	15 (39.5)	
AZI Y	6 (26.1)	28 (73.7)	<0.001
AZI N	17 (73.9)	10 (26.3)	

^*a*^ Y: induced resistance occurred; N: no induced resistance occurred.

No inducible resistance was observed for either CLA or AZI in four patients infected with *M. abscessus* isolates that had the *erm*(41) C28 sequevar, and the sputum conversion rate in this group was 75% (3/4). In contrast, all 32 patients whose isolates carried the *erm*(41) T28 sequevar exhibited a 100% rate of inducible resistance for AZI and an 81.3% rate (26/32) for CLA. The sputum conversion rate for these patients was significantly lower at 18.8% (6/32). *M. abscessus* isolates with the *erm*(41) C28 sequevar are more likely to achieve sputum conversion (*P* = 0.041). Following the Bonferroni correction for multiple comparisons in Table 2, macrolide-inducible resistance was identified as a crucial factor influencing the sputum culture conversion (adjusted *P* < 0.001). The association was further validated through a multivariate logistic regression analysis (Table 3). In 25 cases with *M. massiliense* infection, the sputum conversion rate was 56% (14/25), and all tested isolates contained an *erm*(41) gene with deletions. Additionally, two isolates of *M. massiliense* displayed acquired resistance due to mutations in the *rrl* gene. These isolates were resistant to both CLA and AZI from the outset, and both patients failed to achieve sputum conversion. Acquired resistance in the *rrl* gene was observed exclusively in *M. massiliense* cases in this study. Overall, while the clinical characteristics of *M. massiliense* and *M. abscessus* pulmonary diseases were similar, their treatment outcomes differed significantly, and a much better treatment response was noted in patients infected with *M. massiliense* compared to those with *M. abscessus*.

**TABLE 3:** Factors associated with sputum culture conversion in MABC-PD^*a*^

Characteristics	Multivariate analysis	
	Adjusted OR (95% CI)	*P* value
Gender (female)	1.324 (0.401–4.370)	0.645
Age (years)	1.018 (0.979–1.059)	0.361
Subspecies	6.644 (0.694–63.642)	0.1
Colony (rough/smooth)	1.037 (0.324–3.323)	0.951
Induced drug resistance (Y/N)	10.094 (1.056–96.475)	0.045

^*a*^ Y: induced resistance occurred; N: no induced resistance occurred.

## DISCUSSION

The intrinsic drug resistance of MABC poses significant challenges in treatment, along with the emergence of acquired resistance and inducible resistance to macrolides, which are the primary treatment medications. Consequently, the clinical treatment outcomes of MABC diseases are not ideal. Previous studies indicated that the treatment success rate was relatively higher for *M. massiliense* (>54%), while it is notably lower for *M. abscessus*, ranging from 20 to 40% (13, 25, 26). In this study, we observed an overall sputum culture negative conversion rate of 37.7% for patients with MABC-PD. Consistent with previous findings, the rate of the sputum culture negative conversion was higher in cases of *M. massiliense* compared to *M. abscessus*.

AZI and CLA are potent macrolide antibiotics that inhibit bacterial protein synthesis by blocking peptidyl transferase in the 50S ribosomal subunit, making them effective bacteriostatic agents. Beyond their antibacterial effects, they also reduce inflammatory responses, limit tissue damage, and promote recovery (27, 28). Given the complexities of treating MABC diseases, we investigated the *in vitro* efficacy of these macrolides against MABC and assessed their potential to induce drug resistance, which could shed light on appropriate macrolide drug choices and improve patient outcomes.

Currently, the CLSI does not recommend a critical concentration for drug susceptibility testing (DST) of AZI in MABC. Based on the critical concentrations for CLA that were suggested by the CLSI and supported by existing literature (23, 24), the MIC breakpoints for CLA and AZI in MABC used in our study are both 8 µg/mL. Our study showed that the MICs of *M. abscessus* isolates for AZI are approximately four to eight times higher than those for CLA, consistent with previous studies (29–31). Additionally, the MIC_50_ and MIC_90_ values for AZI were also found to be greater than those for CLA in both *M. abscessus* and *M. massiliense*. Acquired macrolide resistance was observed in only two isolates of *M. massiliense* but none in *M. abscessus* isolates. Both AZI and CLA can induce resistance in *M. abscessus* isolates with the functional *erm*(41) gene. However, we found that resistance induced by AZI developed earlier and occurred more frequently than resistance induced by CLA, which was consistent with previous findings (18, 20, 32). Considering the distribution of MICs, the timing, and the incidence of inducible resistance observed in clinical strains, we proposed that AZI does not exhibit superior anti-*M*. *abscessus* activity compared to CLA *in vitro*. Overall, this study and much data in other studies support that CLA would be a better choice than AZI in MABC disease treatment. Additional clinical research is still required for confirmation.

In the study by Choi et al., the expression of the *erm*(41) gene significantly increased after exposure to CLA compared to AZI in *M. abscessus*, but this was not observed in *M. massiliense* (8). Later, other experts found that both CLA and AZI induced resistance similarly, which means that this determining factor in drug choice is not reliable (18, 20). However, some other studies indicated that AZI induced macrolide resistance earlier than CLA (32), which aligned with our current research. After a comprehensive analysis, we believed the main reason for these inconsistencies was the use of different housekeeping genes as internal reference genes when measuring the transcription levels of the *erm*(41) gene. In the study by Choi et al., the 16S rRNA gene was employed (8), but Schildkraut et al. reported that the expression level of 16S rRNA can decrease in response to macrolide antibiotics and suggested that GAPDH is a more appropriate choice for quantifying the *erm*(41) gene in MABC (20). Thus, we use the GAPDH gene as the internal reference gene in our study, and we also found that the functional *erm*(41) gene was significantly and highly expressed in response to both CLA and AZI exposures in some selected isolates that did not develop CLA-inducible resistance. These findings indicate that evaluating the expression level of this gene may not serve as the most reliable indicator of CLA-inducible resistance. Moreover, it suggests that certain strains that do not show CLA-inducible resistance might utilize alternative mechanisms to circumvent the onset of resistance. Nevertheless, the size of the sample studied is relatively small; thus, comprehensive further research is necessary to clarify these complex interactions and mechanisms.

Our study has several limitations. First, the number of clinical isolates was limited and originated from a single center, which means that while the data are representative, they may not apply to a wider population. Second, most of the MABC isolates were collected through a retrospective analysis from outpatient clinics, which made it difficult to obtain complete follow-up data. While we have included multiple-factor analysis in the statistical section and made necessary adjustments to improve the reliability of our clinical conclusions, we may still face challenges in fully controlling for confounding variables, which is a common issue in retrospective studies. Third, the antibacterial activity and inducible drug resistance observed *in vitro* might not accurately reflect the drug’s effects in patients. Therefore, more data from clinical trials are needed to determine whether CLA is more effective than AZI in treating MABC infections. Finally, in the analysis of *erm*(41) genotypes, patients with the C28 sequevar showed higher rates of sputum culture conversion (*P* = 0.041 according to Fisher’s exact test). However, this finding was based on only four C28-positive cases, which may make the results vulnerable to the influence of outliers and limit the reliability of this association.

In conclusion, while macrolides are effective against MABC, the development of inducible resistance is a significant concern, especially in *M. abscessus* isolates with a functional *erm*(41) gene. Inducible resistance from AZI occurs more rapidly and extensively than that from CLA. Additionally, the clinical culture conversion rate for patients with *M. abscessus* infections is notably lower than for those with *M. massiliense*, highlighting the need for improved treatment strategies.

## MATERIALS AND METHODS

### Study participants

Patients diagnosed with MABC-PD at Beijing Chest Hospital from 2014 to 2020, who completed the entire treatment regimen, were retrospectively enrolled. We collected data on culture conversion, which was defined as obtaining at least three consecutive negative mycobacterial cultures from respiratory samples collected at least 4 weeks apart during antimycobacterial treatment. The date of the first negative culture obtained was considered the conversion date (33). The efficacy of the treatment was evaluated based on sputum culture conversion conducted 12 months after starting treatment.

### Isolate identification and sequencing of *erm*(41) and *rrl* sequevars

The primary isolate, which was cultivated from the sputum collected before starting treatment for MABC-PD, was resuscitated and identified to the species level by homolog gene sequencing. Primers targeting heat shock protein 65 (*hsp65*), the β-subunit of bacterial RNA polymerase (*rpoB*), and the internal transcribed spacer (ITS) between 16S and 23S rRNA were used for the polymerase chain reaction (PCR) (34–36). The PCR amplification was performed in a 20 µL reaction mixture containing 10 µL of 2× Taq Master Mix, 1 µL each of forward and reverse primers (10 µmol/L), 1 µL of DNA template, and 7 µL of double-distilled water. The amplification protocol consisted of an initial denaturation step at 95°C for 10 min, followed by 30 cycles of denaturation at 94°C for 30 s, annealing at 70°C for *hsp65*, 63°C for ITS, or 72°C for *rpoB* for 30 s, and extension at 72°C for 3 min. The final extension step was performed at 72°C for 10 min. The amplified products were then sequenced, and the resulting nucleotide sequences were analyzed using the BLAST tool from the National Center for Biotechnology Information database. If two or more targets identified the same bacterial species, that identification result was considered confirmed. The *erm*(41) and *rrl* genes were sequenced for the identified MABC isolates to check for any mutations.

### Susceptibility testing for CLA and AZI

According to the guidelines of the CLSI (23), the minimum inhibitory concentration (MIC) of CLA and AZI was determined using the broth microdilution method. CLA (C9742, Sigma) and AZI (HY-17506, MCE) were dissolved in DMSO and prepared as stock solutions with concentrations of 25.6 and 12.8 mg/mL, respectively. The tested drug concentration ranges were 0.0156–16 µg/mL for CLA and 0.03125–64 µg/mL for AZI. The culture grown to the logarithmic phase was scraped from the Lowenstein-Jensen (L-J) culture medium and adjusted to a 0.5 McFarland concentration (approximately 10^10 CFU/L) using saline. This suspended bacterial solution was then diluted 200-fold in Mueller-Hinton broth. On the third day, 70 µL of alamarBlue dye (a mixture of 20 µL alamarBlue and 50 µL Tween 80) was added to each well. To evaluate the inducible macrolide resistance, the MICs were measured after incubation periods of 3, 5, 7, 10, and 14 days at 37°C.

### Isolation of RNA from bacterial cultures

Five clinical isolates of *M. abscessus* with the functional *erm*(41) gene (the 28th base is T) that can develop induced resistance were selected. Among these isolates, three exhibited induced resistance to both CLA and AZI, while two isolates developed induced resistance solely to AZI. The induced resistance of selected isolates is shown in Table S1. The reference strain *M.abscessus* ATCC 19977 and the five clinical isolates were cultured at 1/2 MIC of CLA and AZI for 1 and 3 days, respectively. RNA was extracted from the cultures using the Trizol method. For RNA extraction, 10 mL of bacterial culture in the logarithmic growth phase was centrifuged at 4,000 rpm for 10 min to pellet the cells. The pellet was then resuspended in 1 mL of Trizol reagent and thoroughly lysed by repeated pipetting. Next, 200 µL of chloroform was added, and the mixture was vigorously shaken for 30 s. It was then incubated at room temperature for 2 to 3 min or until the solution turned milky white. The mixture was centrifuged at 4°C at 12,000 *g* for 20 min to achieve phase separation. Approximately 400 µL of the upper aqueous phase was carefully collected, taking care to avoid the interphase to prevent DNA contamination. This was then mixed with 600 µL of isopropanol by gentle inversion. The sample was incubated at −80°C for 1 h, followed by centrifugation at 4°C at 12,000 *g* for 10 min to obtain the RNA, which appeared as a gel-like or white pellet at the bottom of the tube. After removing the supernatant, the RNA pellet was washed with 1 mL of ice-cold 75% ethanol by gentle inversion, and then centrifuged at 4°C at 7,500 *g* for 5 min. Ethanol was completely removed by pipetting, and the pellet was allowed to air-dry at room temperature for 5 to 10 min. Finally, the RNA was dissolved in 30 µL of RNase-free water and stored at −80°C.

### Determination of the *erm*(41) expression level

The expression of the *erm*(41) gene was quantified using a one-step reverse transcriptase PCR method, with GAPDH included as a housekeeping gene. The ΔCT method was utilized to assess the differences in *erm*(41) expression on the first and third days after macrolide exposure. Expression levels of *erm*(41) were normalized to the gene expression of GAPDH. The relative mRNA level of *erm*(41) was calculated as the ratio of the drug exposure levels in the 1- and 3-day groups to those in the no-drug-exposure (0-day) group.

### Statistical analysis

All data were analyzed using IBM SPSS Statistics Version 22.0, with the significance level set at *P* < 0.05. Categorical variables are presented as numbers and percentages (%), while continuous variables are reported as median values with interquartile ranges (IQR). Comparisons of categorical variables were conducted using Pearson’s *χ* test when all expected cell frequencies were greater than or equal to 5. In cases where any expected cell frequency was less than 5, Fisher’s exact test was used. To prevent inflation of the type I error from multiple comparisons, the Bonferroni correction was applied, adjusting the significance threshold to 0.05 divided by the number of comparisons (0.05/*n*). For continuous variables, those with a normal distribution were compared using the *t*-test, while those without a normal distribution were analyzed using the Mann–Whitney *U* test.

## Data Availability

The raw data supporting the conclusions of this article will be made available by the authors without undue reservation.

## SUPPLEMENTAL MATERIAL

Figure S1 and Table S1 spectrum.01274-25-s0001.docx Fig. S1: Time-dependent induction of resistance in each isolate. Table S1: Isolates selected for the qPCR analysis of *erm*(41).

ASM does not own the copyrights to Supplemental Material that may be linked to, or accessed through, an article. The authors have granted ASM a non-exclusive, world-wide license to publish the Supplemental Material files. Please contact the corresponding author directly for reuse.
