# Supplementary material for: Culture conversion and macrolide resistance in Mycobacterium abscessus complex pulmonary disease
Source: Microbiol Spectr. 2025 Sep 2;13(10):e01274-25. doi: 10.1128/spectrum.01274-25 (PMC12502594; doi:10.1128/spectrum.01274-25)
Supplement: Figure S1 and Table S1 — Fig. S1: Time-dependent induction of resistance in each isolate. Table S1: Isolates selected for the qPCR analysis of erm(41). [file spectrum.01274-25-s0001.docx]

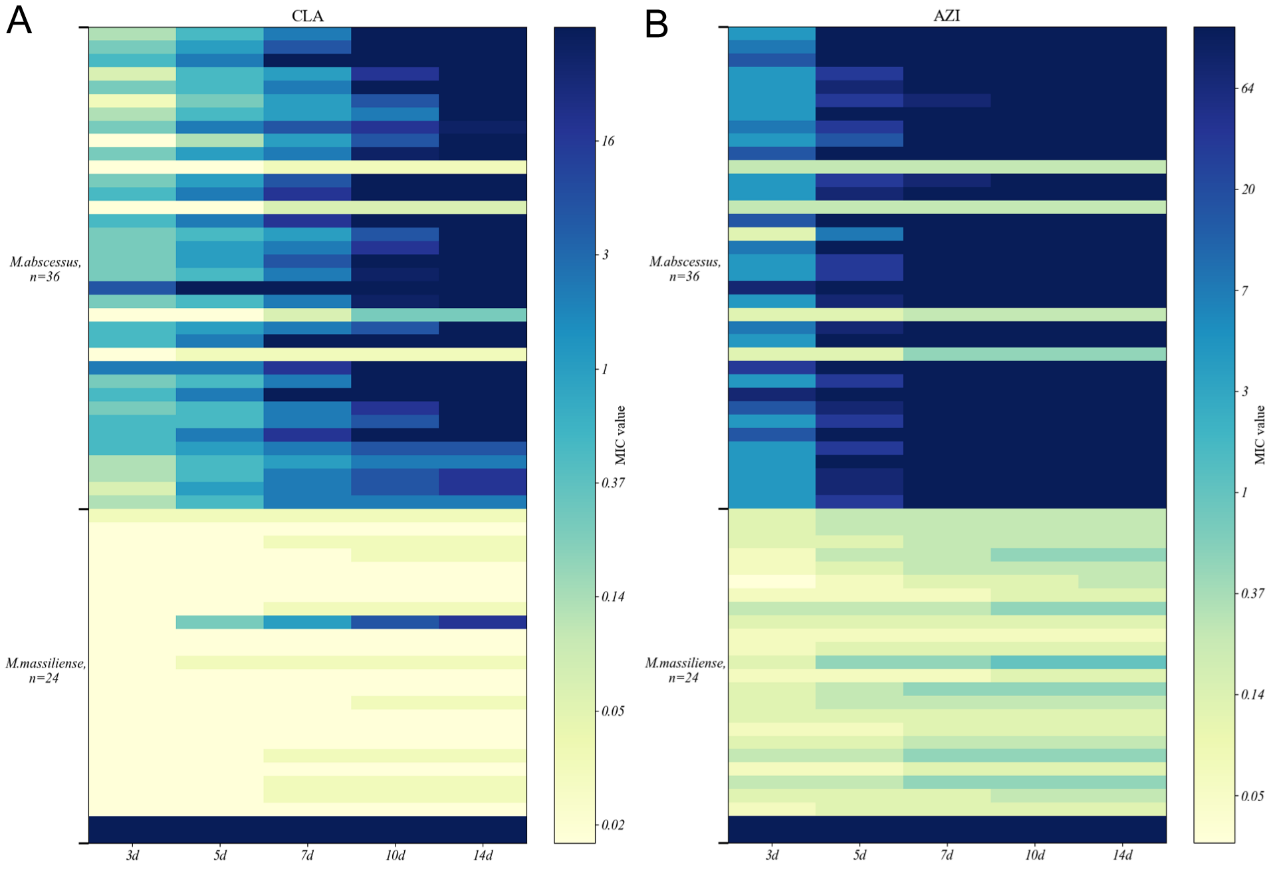


Supplementary Figure 1. Time-dependent induction of resistance by CLA and AZI in *M. abscessus* and *M. massiliense* isolates. A. The change of CLA MIC value in each isolate at different incubation time. B. The change of AZI MIC value in each isolate at different incubation time.

| Sample ID | erm(41) Position 28 | Culture conversion | CLA Inducible Resistance (induction time) | AZI Inducible Resistance (induction time) |
| --- | --- | --- | --- | --- |
| ATCC 19977 | T | NA | Yes (14th day) | Yes (10th day) |
| 690 | T | Yes | Yes (7th day) | Yes (5th day) |
| 801 | T | No | Yes (14th day) | Yes (5th day) |
| 2689 | T | Yes | Yes (14th day) | Yes (7th day) |
| 1642 | T | No | No | Yes (7th day) |
| 3252 | T | No | No | Yes (5th day) |

Supplementary Table 1. Isolates selected for qPCR analysis of *erm*(41).

NA: not applicable.
